# Urinary Incontinence in Competitive Women Powerlifters: A Cross-Sectional Survey

**DOI:** 10.1186/s40798-021-00387-7

**Published:** 2021-12-07

**Authors:** Lolita Wikander, Marilynne N. Kirshbaum, Nasreena Waheed, Daniel E. Gahreman

**Affiliations:** 1 College of Health and Human Sciences, Charles Darwin University, Casuarina, NT Australia; 2 Research and Innovation, Charles Darwin University, Casuarina, NT Australia; 3 College of Nursing and Midwifery, Charles Darwin University, Casuarina, NT Australia

**Keywords:** Women’s health, Pelvic floor, Urinary incontinence, Resistance training

## Abstract

**Background:**

Urinary incontinence (UI) can negatively affect a woman’s quality of life, participation in sport and athletic performance. The objectives of this study were to determine the prevalence of UI in competitive women powerlifters; identify possible risk factors and activities likely to provoke UI; and establish self-care practices.

**Methods:**

This international cross-sectional study was conducted using an online survey completed by 480 competitive women powerlifters aged between 20 and 71 years. The Incontinence Severity Index (ISI) was used to determine the severity of UI.

**Results:**

We found that 43.9% of women had experienced UI within the three months prior to this study. The deadlift was the most likely, and the bench-press the least likely exercise to provoke UI. ISI scores were positively correlated with parity (*τ* = 0.227*, p* < 0.001), age (*τ* = 0.179*, p* < 0.001), competition total (*τ* = 0.105, *p* = 0.002) and body mass index score (*τ* = 0.089, *p* = 0.009*)*. There was no significant correlation between ISI and years strength training (*τ* = − 0.052, *p* = 0.147) or years powerlifting (*τ* = 0.041, *p* = 0.275). There was a negative correlation between ISI score with having a pelvic floor assessment (*η* = 0.197), and the ability to correctly perform pelvic floor exercises (*η* = 0.172).

**Conclusion:**

The prevalence of UI in this cohort was at the upper limit experienced by women in the general population. Women who had undergone a pelvic floor examination or were confident in correctly performing pelvic floor exercises experienced less severe UI.

**Supplementary Information:**

The online version contains supplementary material available at 10.1186/s40798-021-00387-7.

## Key Points

- The prevalence of UI in this cohort of competitive women powerlifters was at the upper limit of the range of UI experienced by women in the general population.
- Participants who had undergone a pelvic floor examination or who were confident in their ability to correctly perform pelvic floor exercises experienced less severe UI.
- Incontinent participants employed numerous strategies related to their setup, bracing, technique, breathing and the activation of their pelvic floor to minimise leakage of urine during training and competition.

## Introduction

The prevalence of urinary incontinence (UI) in women, defined as a complaint of involuntary loss of urine [1], is thought to range between 25 and 45% [2]. The prevalence of UI in high impact sports such as trampolining is reported to be 80% while the prevalence of UI in non-impact activities, such as yoga and Pilates, has been found to range between 5.56% [3] and 25.9% [4]. Experiencing UI has the potential to negatively affect participation in sport and exercise [5, 6], athletic performance [7, 8] and quality of life [9]. The most common risk factors for UI in women are age, a high Body Mass Index (BMI), parity and delivery type [2]. In addition to risk factors for UI in the general population, sport and exercise related risk factors such as load [8, 10, 11], body position [10] and training to fatigue may result in short term pelvic floor muscle fatigue [12, 13], raise intrabdominal pressure or provoke UI in women who participate in sport [14]. Research investigating the direct association between heavy lifting, pelvic floor health and UI is limited [15]. Hence, knowledge gaps prohibit firm conclusions regarding the effect of strenuous sporting activities on the incidence of pelvic floor disorders [16].

Most commonly UI is classified as stress (complaint of involuntary loss of urine on effort or physical exertion including sporting activities, or on sneezing or coughing [1]), urgency (complaint of involuntary loss of urine associated with urgency [1]) or mixed UI (complaint of both stress and urgency UI [1]). The term athletic incontinence is not an officially recognised term [17] but a term used either generally in the literature to describe UI experienced during athletic activity [18] or more specifically to describe UI experienced by otherwise continent young nulliparous women only during training and competition [19]. More recently, the term athletic incontinence has been used to describe UI experienced during exercise by otherwise continent women of any age or parity [11, 17]. While stress UI, as suggested by its definition, can be provoked by sporting activities that this study does not focus on the mechanical classifications of UI but the contexts in which UI is likely to occur (everyday activities, training or competition or both).

In addition to quality of life and participation in sport and exercise, UI has the potential to affect athletic performance [7, 8]. UI is most problematic in sports such as rhythmic gymnastics [7], weightlifting and powerlifting [8] where women athletes wear tight clothing and compete as an individual rather than in a team [20]. While women athletes are unlikely to seek medical attention for their UI or disclose the health condition to their coaches, they do believe that UI should not be left to them to manage alone [21]. Professionals who support women athletes therefore need to be aware of the prevalence of UI and its implications [22] to ensure early identification of athletes who are at risk, enabling early, quality treatment and interdisciplinary clinical collaboration [23].

This study investigated UI in competitive women powerlifters. Powerlifting is a strength-based sport where participants aim to lift maximum amounts of weight for a single repetition in the squat, bench press and deadlift. Our study aimed to determine if load, body position and fatigue influenced the likelihood of UI in competitive women powerlifters during training and competition. Secondly, our study aimed to determine the prevalence of UI in competitive women powerlifters and the relationship between risk factors and their Incontinence Severity Index (ISI) score, a validated tool used to assess the severity of urinary incontinence in epidemiological surveys [24]. Thirdly, we aimed to determine participants’ confidence in performing a pelvic floor contraction and finally, our study sought to identify activities that provoked UI and the self-care practices participants engaged in to manage leakage of urine during training and competition.

## Methods

### Study Design

This research was an online cross-sectional survey (Additional file 1) developed based on feedback from a pilot study which investigated the prevalence of UI in predominantly Australian women powerlifters [25]. The pilot study only included a limited number of questions focusing on sport related risk factors and failed to investigate everyday risk factors such as parity and BMI. The pilot survey included an open-ended question where some participants chose to include feedback and suggestions for further research. The survey in this study was developed based on feedback from participants and suggestions from reviewers of the pilot study. Survey questions in the current study focused on the context in which UI occurs (everyday activities, training or competition or both) and the exercises most likely to provoke UI rather than the traditional classifications of UI such as stress, urgency and mixed incontinence [18]. Additionally, the survey utilised the Incontinence Severity Index (ISI); a validated tool that quantifies the frequency and severity of UI [26]. The ISI was chosen as it has been deemed a useful tool for the assessment of the severity of UI in epidemiological studies [24]. The ISI is highly sensitive to change seen with treatment [27] and has good criterion validity against 24-h pad tests [27]. Finally, the ISI has been found to correlate highly with other validated questionnaires for assessing the severity of UI such as the International Consultation on Incontinence Questionnaire-Urinary Incontinence Short Form [28].

In this survey, UI was defined as an ISI score > 0. All women who had experienced leakage of urine, regardless of context, were considered incontinent when determining the lifetime prevalence of UI. Athletic incontinence was defined as UI during training or competition in otherwise continent women of any age or parity [17]. All women, continent and incontinent, were included when determining the correlation between ISI score and possible risk factors. Open-ended questions were used to collect data pertaining to activities that exacerbated UI in this cohort and to identify self-care practices used to prevent, reduce or conceal UI in training and competition.

### Participants

Women powerlifters aged between 20 and 89 years competing in three lift meets at a local, national or international level were eligible to participate. The lower end of the age range was selected as it corresponded to the lowest age group in the pilot study, and the higher end of the age range reflected the maximum age group commonly used in the recording of national and international records [29, 30]. The survey was prefaced by a research participant information sheet, which provided assurance that the questionnaire was anonymous, and participation was voluntary. In addition, participants were informed that they could withdraw from the study before submitting their responses by closing the browser. Ethics approval was obtained from a relevant Human Research Ethics Committee at an Australian University, and the study was performed in accordance with the standards of ethics outlined in the Declaration of HELSINKI and the National Statement on Ethical Conduct in Human Research 2007 (Updated 2018) [31].

### Procedure

An online survey tool (Qualtrics, Provo, Utah & Seattle, WA, USA) was used to collect data. The survey was circulated through Facebook and emailed to intermediates such as powerlifting associations and gymnasiums located in English speaking countries such as the United Kingdom, the United States of America, Australia, Canada and New Zealand.

Participants were recruited by two main methods. Firstly, a list of intermediates was generated by conducting a Google search for ‘English speaking countries’ and combining the results from this search with the term ‘Powerlifting’. A recruitment email was then sent to the resulting list of contacts. The second method used Facebook to recruit participants. Facebook groups that were likely to contain competitive women powerlifters were identified, contacted and asked to publish or email a link to the survey. Paid advertisements, using a dedicated Facebook page, also targeted women from English speaking countries who met the selection criteria.

### Statistical Analyses

We analysed the data using the Statistical Package for the Social Sciences (SPSS 27.0 Inc., Chicago, IL). Central tendency and dispersion were reported as means ± standard deviation (SD), and descriptive data were presented in percentages and the absolute numbers. The relationship between risk factors and ISI score was investigated using Kendall’s tau-b with the level of significance set at *p* ≤ 0.05 for all analyses. The association between pelvic floor examination or confidence in performing a pelvic floor contraction with ISI score was assessed by Eta correlation coefficient, and the results were reported in *η*.

## Results

The survey received 778 responses. However, 154 participants exited before completing the survey, and their data were excluded from this study. A further 142 responses were removed as respondents indicated that they did not compete in full powerlifting meets (all three lifts). Additionally, 2 respondents were under the age of 20 years and therefore excluded. The data of the remaining 480 participants were analysed and reported in this paper.

Participants of this study were competitive women powerlifters (Age: 35 ± 10 years; Height: 1.64 ± 0.07 m: Body Mass: 74 ± 18 kg; and BMI: 27.61 ± 6.01 kg/m^2^) who had an average three lift competition total of 322 ± 74 kg. The competitive women powerlifters in this study had participated in some form of strength training for 5.47 ± 4.28 years and had been powerlifting for 2.85 ± 1.94 years. In this study, 36% (*N* = 173) of women had given birth. In the group of 173 women, who had given birth 69.4% (*N* = 120) had delivered vaginally, 21.4% (*N* = 37) by caesarean section, and 9.2% (*N* = 16) women had experienced both vaginal and caesarean births.

Approximately, 48.8% (*N* = 234) of participants in this study reported experiencing UI at some point in their lifetime, and 43.9% (*N* = 211) had experienced UI in the three months prior to the study. Athletic incontinence had been experienced by 23.1% of women (*N* = 111). We found that 17.9% of women (*N* = 86) had been continent before commencing powerlifting but now experience UI in training or competition but not during everyday activities (Type 1 athletic incontinence) [17]. Furthermore, 5.2% of women (*N* = 25) were incontinent before commencing powerlifting but are now continent during everyday activities while continuing to experience UI during training or competition (Type 2 athletic incontinence) [17].

UI was experienced in competition during maximum lift attempts by 30.6% (*N* = 147) of participants, in training during maximum lift attempts by 40.4% (*N* = 194) of participants, during sumo deadlifts by 12.5% (*N* = 60) of participants, and in high repetition sets by 35.2% (*N* = 169) of participants. Interestingly, 79.3% (*N* = 134) of the women experiencing UI during high repetition sets indicated that UI was only an issue if the sets were heavy and 64.5% (*N* = 109) stated that UI was worse at the end of sets.

Most women reported being confident (46.5%, *N* = 223) or very confident (25.2%, *N* = 121) in their ability to correctly perform a pelvic floor contraction, and 14.2% (*N* = 68) had undergone a pelvic floor assessment. In the subset of women who had experienced UI in some point in their life 47.4% (*N* = 111) reported being confident in their ability to perform pelvic floor exercises, 19.2% (*N* = 45) were very confident in their abilities, and 20.9% (*N* = 49) had undergone a pelvic floor assessment. There was a small negative correlation between ISI score with having had a pelvic floor assessment, (*η* = 0.197), or confidence in their ability to perform pelvic floor exercises, (*η* = 0.172).

**Fig. 1:**
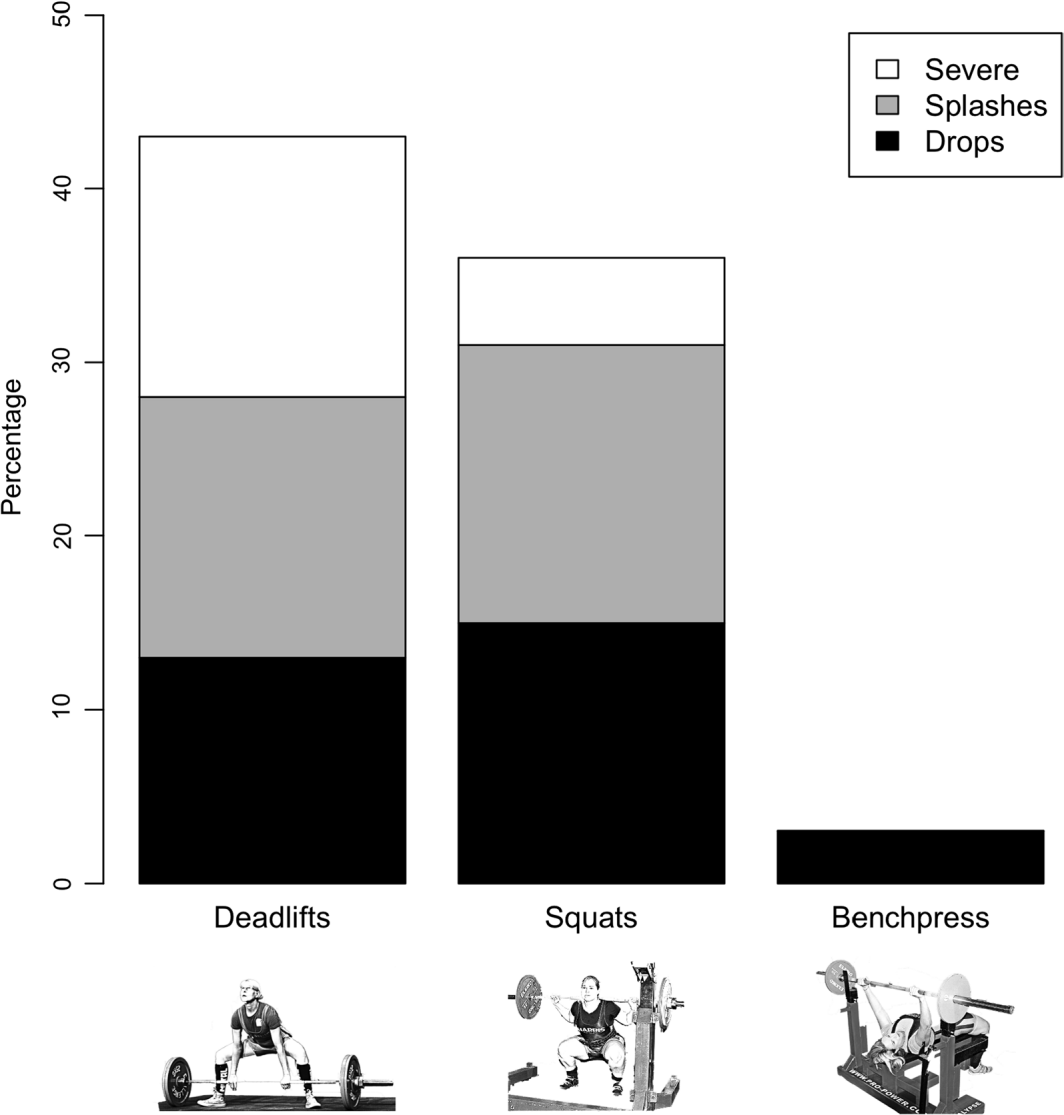
The percentage of competitive women powerlifters who experience urinary incontinence during powerlifting lifts and the severity of leakage experienced during lifts

Deadlifts were most likely to provoke leakage while the bench press provoked only very mild leakage in very few women. Figure 1 illustrates the percentage of women experiencing UI and the severity of UI during the three powerlifting lifts.

The correlation between ISI score and age, number of births, competition total, years strength training, years powerlifting and BMI are presented in Table 1. These correlations are reported in all participants of this study and in subcategories of women who had or had not given birth.

**Table 1:** Correlations between Incontinence Severity Index score and possible risk factors for urinary incontinence in all participants, participants who have given birth or participants who have not given birth

	All participants (*N* = 480)	Given birth (*N* = 173)	Not given birth (*N* = 307)
Age (years)	*τ* = 0.179, *p* < 0.001	*τ* = 0.066, *p* = 0.246	*τ* = 0.094, *p* = 0.035
Number of births	*τ* = 0.227, *p* < 0.001	*τ* = 0.179, *p* = 0.005	N/A
Competition total (kg)	*τ* = 0.105, *p* = 0.002	*τ* = 0.046, *p* = 0.409	*τ* = 0.153, *p* < 0.001
Years strength training	*τ* = 0.052, *p* = 0.147	*τ* = − 0.019, *p* = 0.744	*τ* = 0.073, *p* = 0.113
Years powerlifting	*τ* = 0.041, *p* = 0.275	*τ* = − 0.002, *p* = 0.975	*τ* = 0.033, *p* = 0.486
BMI (kg/m^2^)	*τ* = − 0.089, *p* = 0.009	*τ* = − 0.043, *p* = 0.445	*τ* = − 0.126, *p* = 0.004

BMI, Body Mass Index

Reported activities, in addition to maximum effort lifts, high repetition sets, heavy sets and wearing a belt, that provoked UI are summarised in Tables 2 and 3 lists self-care practices used by incontinent participants to prevent, reduce or conceal UI.

**Table 2:** Activities that provoked urinary incontinence

Powerlifting lifts	Physical activity	Oral intake	Non-lifting related
Straining	Box squats	Amount of water drunk before lifting	A full bladder
Over tight belt	Cardio	Caffeine	Anxiety
Bracing poorly or too much	Skipping/jumping	Beta-alanine	Certain times of the menstrual cycle
Belt position	Focused heavy core work	Alcohol	Fatigue
At sticking point before lock out in deadlift	Front squats		Coughing or sneezing
Grinding out a rep	Heavy cleans		Crohn’s disease flair up
Deadlifting with a rounded back	Wrestling		Illness
Poor set up during deadlifts			When getting close to the toilet
Incorrect breathing during bracing			Aggressive sex
Lockouts			Having recently urinated
			During episodes of low back pain
			Tight hips/adductors
			Obesity
			Laughing
			Not doing enough pelvic floor exercises
			Getting closer to home
			Constipation
			Dehydration

**Table 3:** Self-care practices competitive women powerlifters engage in to prevent, reduce or contain leakage of urine

Bracing related	
Brace against the belt Bracing abs and pulling up pelvic floor Bracing my core correctly helps Change the way I brace with my belt Correct bracing Do a Kegel as part of my bracing Focus on bracing with each heavy rep Finding the right bracing in my lifts Focus on bracing properly I'm just starting Kegels and trying to brace better Practicing bracing and breathing in a way so I don’t feel as much pressure on my pelvic floor Proper bracing and core control Re check belt position, ensure proper bracing technique Tighten pelvic floor before bracing for lifts Try to work on bracing and holding the tightness through the lift Be aware of bracing technique Working on bracing through entire stomach I ensure that after a preparation and testing phase that I have a period of time training volume without a belt and with higher reps without breath holding and bracing Kegel before lift. I have been experimenting with bracing, not taking in a full belly of air as is recommended. That second technique might be helping a little, though I worry I am not as effective at bracing as other women	

Preparation/setup related	
Blow before you go, use of my exhale I learnt how to let out a bit of air (very slight) from my abdominal area during bracing, before I begin the descent of my squat, and that seems to help with the leakage Pelvic floor lift prior to the lift and not trying to hold it too much A dynamic warmup and a post workout stretching routine	

Pelvic floor related	
Active pelvic floor engagement before lift Before a lift I try to "lift" my pelvic floor with the same movement you do when you close the zipper on a pair of tight jeans I try not to do too intense of a PF contraction Bracing pelvic floor Control pelvic floor Engage my pelvic floor as much as possible Focus on tensing pelvic floor during the lift I do pelvic floor, and brace correctly I will concentrate on lifting pelvic floor, exhaling on effort and have a break from barbell work if needed to train and strengthen pelvic floor I squeeze my pelvic floor muscles as hard as I can and I wear sanitary pads Try to focus on pulling pelvic floor up instead of bearing down during lifts Maintain focus on pelvic region during high volume sets Being more conscious of engaging pelvic floor before anything else when I brace Pelvic floor exercises My coach has programmed daily core and pelvic floor engagement and strengthening movements Including a proper pelvic floor warmup before training has vastly improved my bladder control Engaging my core and pelvic floor before lifting Pelvic floor holds	

Technique/form/breathing related	
Spreading the load when performing heavy lift Breathing out on lifts Breathing techniques Exhale during concentric part of lift Focus on technique, sometimes helps because I feel like I can control it on single lifts I have several physio cues around ribcage positioning, bracing, and different vaginal muscle cues Improving form on my lifts and bracing correctly is reducing the incidences of leakage I'll concentrate on good form, breathing and bracing Mindful lifting Thinking about pulling my bellybutton in when lifting heavy	

Other training related	
Not wearing a belt Train beltless if I'm feeling particularly leaky Avoid wearing my belt until I have to Going to the bathroom quite often/between sets during training Drink less water on heavy days or during competitions Train dehydrated on lower leg days My trainer suggested kettle bell swings Take spare underwear to gym and competitions	

General	
Always wear a pad Wear incontinence underwear Wearing a super tampon Diva cup (pressure on bladder wall to support muscles) Use a moon cup See a women’s health physio Deep/superficial core exercises I try to observe good bowel emptying practices like not straining to poo Reducing caffeine I currently am using Birthfit training to work on my pelvic floor and stop to pee all the time I try to not hold my pee in for long periods I try not to not force myself to empty my bladder whenever convenient in anticipation of busy hours Being well rested Yoga Use tens Stretching and massage Researching what other women do Powerlifting Not laughing Avoiding sit ups/jumping Learning how to relax pelvic floor muscles Dead bug exercise Using a pelvic weight Menopause has helped	

## Discussion

### Prevalence of UI

The prevalence of UI in this study (43.9%) was in the mid-range of UI experienced by athletes (5.56–80%) [3, 32] and at the upper limit experienced by women in the general population (25–45%) [2]. Furthermore, the prevalence of UI in this cohort was higher than that found in similar studies investigating UI in competitive women weightlifters (31.9%) [11]. Skaug et al., however, reported an even higher prevalence of UI (50%) in a combined cohort of competitive Norwegian weightlifters and powerlifters [8]. Out of the women who reported experiencing UI in their study, 78.9% were powerlifters, and 21.1% were weightlifters. This may imply that the prevalence of UI was much higher in Norwegian powerlifters than weightlifters. However, the number of powerlifters in their study was more than double the number of weightlifters [8]. When adjusted, relative to the number of participants in each cohort, UI was experienced by 56% of powerlifters and 36% of weightlifters. After adjusting for the number of participants, their findings still support our results that UI is experienced by more powerlifters than weightlifters. The exact mechanism for greater UI in powerlifters compared to weightlifters is not fully known. However, it is possible that powerlifters experience greater UI in training and competition as they lift greater loads. Indeed, our findings suggest that the higher prevalence of UI in powerlifters is primarily due to a higher prevalence of athletic incontinence.

The prevalence of athletic incontinence was higher in this study (23.1%) than in similar studies examining athletic incontinence in weightlifters (16.2%) [11]. It is noteworthy, however, that the main difference was observed in type 1 athletic incontinence: weightlifters 8.4% [11], powerlifters 17.9%. This finding indicates that previously continent women who commence powerlifting are more likely than weightlifters to experience UI during training and competition but are not necessarily more likely to be incontinent during everyday activities. While UI during exercise may deter participation, athletic incontinence may be tolerated by women if leakage is minimal, only occurs infrequently, or at maximal effort, and they remain continent during everyday activities.

### Correlations

Parity, age and BMI were the risk factors in the general population that correlated with UI in this study [2]. Competition total was the only sport specific risk factor found to significantly correlate with participants’ ISI scores. To further explore the correlation between ISI and general and sport specific risk factors, we divided our cohort into women who had given birth and women who had not given birth (Table 1). Interestingly, age, BMI and competition total were significantly correlated with ISI scores only in women who had not given birth. Giving birth is possibly the greatest risk factor for UI in women, hence, some younger women, who had given birth, also experienced UI. This could explain why UI in the cohort of women who had given birth was not correlated with age. Contrary to our findings, Skaug et al. performed a multivariate logistic regression and found that only BMI had a significant positive association with stress UI [8]. It is difficult to explain the discrepancy in the findings, however, differences in participants and assessment tools may explain the different results. For example, Skaug et al. recruited a more homogenous sample of nationally and internationally competitive male and female powerlifters and weightlifters who competed at a higher level than our participants [8].

### Sport Related Factors

Data pertaining to sport specific factors thought to contribute to the provocation of UI were collected based on the feedback from the pilot study [25] and the relevant literature. The three sport related factors of interest were: load, body position and fatigue.

#### Sport Related Factors—load

Our results showed that participants were more likely to experience UI during heavy lifts. Indeed, participants who lifted the most weight in competition experienced the most severe UI. The deadlift is the lift where powerlifters are likely to lift the greatest amount of weight, and correspondingly, the deadlift was the lift most likely to provoke UI (42.5%). Our results supported the findings by Skaug et al., who also reported that the deadlift was the lift most likely to provoke UI in competitive weightlifters and powerlifters [8]. The squat was the second most likely competition lift to provoke UI in our cohort of powerlifters. While 36.3% of participants in our study reported experiencing UI during squats, comparable studies reported that 23% of weightlifters [11] and 12.6% of CrossFit participants [17] experienced leakage of urine while squatting. Squats are possibly less likely to provoke UI in weightlifters and CrossFit participants due to a difference in the amount of weight lifted, and the number of sets and repetitions performed during training or competition. The primary goal of CrossFit training is to develop fitness as well as strength, and while CrossFit participants may occasionally work up to a One Repetition Maximum (1-RM), this is not the exclusive aim of the sport. Weightlifters are more likely than CrossFit participants to perform sets of squats with heavier weights to develop their strength rather than endurance. While powerlifters also focus on gaining strength, they are likely to train with heavier weights than weightlifters and perform sets with fewer repetitions to lift the maximum weight possible during a single repetition. Despite small variations in squat technique between powerlifters, weightlifters and CrossFit participants, it is possible that the prevalence of UI is influenced by the total weight lifted. We note however, that while lifting a progressively heavier weight has been shown to increase intrabdominal pressure [10], it is more difficult to demonstrate that lifting heavy weights provokes UI [15, 33].

Interestingly, some of the powerlifters in this study and the pilot study [25] reported that with progress in training capacity, they were able to lift heavier weights without leaking. An increase in UI threshold and urinary leakage were only experienced at near or above their previous maximum lifts. In other words, they were still incontinent, just at a higher lifting weight.

#### Sport Related Factors—Body Position

In addition to the total amount of weight lifted, some women reported that other factors such as body position during lifts provoked UI. Body position while lifting a weight has been found to influence the amount of intra-abdominal pressure [10], a phenomenon thought responsible for leakage during physically exerting actions. In our study, the bench-press, in comparison to the two standing lifts, was very unlikely to provoke urinary leakage. This finding supports the recommendation of low impact activities that do not place downwards pressure on the pelvic floor when the aim is to prevent UI during sport and exercise [34]. While the bench-press is usually the lift where the least amount of weight is lifted, women still lift a significant amount of weight with some women lifting more than double their own body weight.

Body position during the deadlift was also problematic for some women who reported that sumo deadlifts, where the lifter has a wider stance compared to the conventional deadlift, were more likely to provoke UI. The possibility that sumo deadlifts provoke UI in some powerlifters has been identified in a previous study [25]. Wearing a lifting belt and exercises that put further pressure on the abdominal area such as sit ups and front squats were reported to be problematic for some women.

#### Sport Related Factors—Fatigue

Pelvic floor muscle fatigue can exacerbate UI, especially stress UI [14]. Our participants reported that fatigue provoked UI if they were not well rested before a training session, their sets were long and/or heavy and if individual lifts were difficult and slow. A little over a third of the women experienced leakage during high repetition sets, especially at the end of heavy sets, indicating that not only the amount of weight lifted and body position but also fatigue played a part in the provocation of UI in this cohort of powerlifters. One explanation why fatigue contributes to UI in women athletes is that women training at a competitive level may have a stronger pelvic floor than non-trained women but less pelvic floor muscle endurance [35].

### Activities Found to Provoke UI and Self-care Activities

Furthermore, participants in this study provided an extensive list of activities that they felt provoked UI (Table 2). The activities specific to powerlifting that provoked UI were focused on bracing, lifting technique and how they wore their belt. Outside powerlifting training jumping or skipping movements were the most problematic activities. Research with CrossFit participants have found that the cardio component of training sessions such as running and skipping is more likely to provoke UI in women than lifting weights [36, 37]. Many women were conscious of the amount of fluid that they consumed prior to lifting, and some would limit fluids prior to training or competition or even train dehydrated. Minimising fluid intake to manage UI during physical activity had previously been reported [36, 38].

Participants in this study also provided an extensive list of training and competition related strategies utilised to prevent, reduce or contain leakage. Many self-care strategies revolved around warming up for training or competition, correct set up, activation of the pelvic floor and focusing on technique and bracing. A more detailed list of responses is available in Table 3. It is noteworthy that there is very little or no research specific to powerlifting and strategies to prevent or minimise leakage of urine during training or competition. The women were therefore relying on their own experience, the internet or expert opinion rather than evidence-based practice. Practice is preceding research in this area and consequently coaches, trainers, physiotherapists and medical professionals who support women powerlifters are largely relying on trial and error and research that is not powerlifting or even sport specific. While our study is descriptive and cannot assign causation, it will increase the knowledge of UI in competitive women powerlifters and help form a foundation for future research.

### Pelvic Floor Exercises and Pelvic Exams

Most of the participants in this study reported that they were confident in their ability to perform pelvic floor exercises. It has been shown, however, that depending on the population being studied as many as 15.1–65% of women do not know how to correctly perform a pelvic floor contraction [39–41]. A total of 68 participants in this study had undergone a pelvic floor examination and therefore, most women had not had their ability to generate an effective, timely pelvic floor contraction assessed. Having undergone a pelvic floor examination or being confident in their ability to perform pelvic floor exercises were both negatively correlated with ISI scores suggesting that these subgroups of women experienced less severe UI. This finding supports the recommendation that athletes undergo a pelvic floor assessment and have their ability to correctly perform pelvic floor exercises confirmed [7].

### Limitations

While valuable information was provided by a large cohort of women powerlifters, it is important to acknowledge that this study had some limitations. Firstly, our study used a cross sectional design to identify the association between risk factors and UI. This design fails to assign and investigate causation. Secondly, it was not possible to avoid potential survivor bias when considering the relationships between UI and powerlifting. Secondly, the recruitment of participants through social media and by email may have introduced further bias as women who have experienced UI may have been more interested in completing the survey. The women also relied on recall when answering the survey questions, as opposed to being evaluated based on an objective measurement. Despite these limitations, this study builds on the knowledge obtained in the pilot study and greatly increases understanding within this niche area. The results from this study form an important basis for future studies in an under-researched topic.

## Conclusion

The amount of weight lifted, body position and fatigue were powerlifting related risk factors that participants in this study identified as potential triggers for leakage of urine during training or competition. Parity, BMI and age were possible risk factors for UI that our participants shared with women in general; while competition total was identified as a possible lifting sports related risk factor. The participants in this study had a greater prevalence of type one athletic incontinence compared to other strength sports such as weightlifting. Participants who had undergone a pelvic floor examination or who were confident in their ability to correctly contract their pelvic floor experienced less severe UI. Finally, this study provided valuable information relating to the prevalence of UI, which powerlifting lifts and activities are more likely to provoke leakage of urine and how urinary leakage is managed by women powerlifters during training and competition.

## Supplementary Information

**Additional file 1.** Urinary incontinence in competitive women powerlifters survey

## Data Availability

Data available on request.
