# Supplementary material for: Urinary Incontinence in Competitive Women Powerlifters: A Cross-Sectional Survey
Source: Sports Med Open. 2021 Dec 7;7:89. doi: 10.1186/s40798-021-00387-7 (PMC8651931; doi:10.1186/s40798-021-00387-7)
Supplement: Supplementary file 1 — Additional file 1. Urinary incontinence in competitive women powerlifters survey [file 40798_2021_387_MOESM1_ESM.pdf]

Title page - Electronic supplementary material

Article title

Urinary incontinence in competitive women powerlifters: A cross-sectional survey

Journal name

Sports Medicine - Open

Author names

Lolita Wikander[a], Marilynne N Kirshbaum[b], Nasreena Waheed[c], Daniel E Gahreman[a]

Author affiliations

[a]College of Health and Human Sciences, Charles Darwin University, Northern Territory, Australia

[b]Research and Innovation, Charles Darwin University, Northern Territory, Australia

[c]College of Nursing and Midwifery, Charles Darwin University, Northern Territory, Australia

Corresponding author: Lolita Wikander

Email: lolita.wikander@yahoo.com

Urinary incontinence in competitive women powerlifters survey

Q1 Do you currently compete in three lift powerlifting meets at a local, national or international level?
Q2 Please nominate your year of birth
Q3 How many years have you participated in ANY type of strength/weight training?
Q4 How many years have you been powerlifting?
Q5 What is your current body weight in kilograms?
Q6 What is your height in centimeters?
Q7 What is your best ever three lift competition total in kilograms?
Q8 Have you ever had a pelvic floor assessment?
Q9 How confident are you in your ability to perform pelvic floor exercises?
Q10 Have you ever given birth?
Q11 Number of births?
Q12 Did the births occur before or after you started Powerlifting?
Q13 Were your births vaginal or cesarean sections?
Q14 Were any of your vaginal births instrumental births?
Q15 What type of instrumental births were they? If you have had both types please select both.
Q16 Did your instrumental birth occur before or after commencing powerlifting?
Q17 Have you had any urogenital (reproductive organs or urinary system) surgery?
Q18 Did you have your surgery before or after commencing powerlifting?
Q19 Do you or have you ever suffered from urinary incontinence?
Q20 Did you suffer from any form of urinary incontinence before powerlifting?
Q21 Have your symptoms improved, stayed the same or gotten worse as your lifting age has increased?
Q22 When is your incontinence worse?
Q23 Do you experience urinary incontinence during training?
Q24 Do you experience urinary incontinence outside training time?
Q25 Do you ever experience urinary incontinence when competing?
Q26 Do you ever experience urinary incontinence during a max effort lift in competition?
Q27 Do you ever experience urinary incontinence during a max effort lift in training?
Q28 Do you ever experience incontinence during high repetition sets?
Q29 Is urine leakage more likely to occur at the end of the set?
Q30 Which activities cause urine leakage?
Q31 Do sumo deadlifts cause more urine leakage than conventional deadlifts?
Q32 Does wearing a belt make your incontinence worse?
Q33 Does anything else make your incontinence worse?
Q34 What practices do you engage in to control the possibility of urine leakage?
Q35 Have you experienced any urine leakage in the last three months?
Q36 How much urine do you lose each time?
Q37 Are there any other comments you would like to make?
